# Diabetes Mellitus Risk Prediction in the Framingham Offspring Study and Large Population Analysis

**DOI:** 10.3390/nu17071117

**Published:** 2025-03-24

**Authors:** Masumi Ai, Seiko Otokozawa, Ching-Ti Liu, Bela F. Asztalos, Julia Maddalena, Margaret R. Diffenderfer, Giuseppina Russo, Nuntakorn Thongtang, Michael L. Dansinger

**Affiliations:** 1 Cardiovascular Nutrition Laboratory, Human Nutrition Research Center on Aging at Tufts University, Tufts University School of Medicine, Boston, MA 02111, USA; seiko.otokozawa@gmail.com (S.O.); bela.asztalos7@gmail.com (B.F.A.); margaret.diffenderfer@bostonheart.eurofinsus.com (M.R.D.); giuseppina.russo@unime.it (G.R.); nuntakorn.thongtang@gmail.com (N.T.); mdansinger@gmail.com (M.L.D.); 2 Department of Insured Medical Care Management, Institute of Science Tokyo, Tokyo 113-8519, Japan; 3 Department of Public Health, Sapporo Medical University School of Medicine, Sapporo 060-8556, Japan; 4 Department of Biostatistics, Boston University School of Public Health, Boston, MA 02118, USA; ctliu@bu.edu; 5 Framingham Heart Study, Framingham, MA 01702, USA; 6 Boston Heart Diagnostics, Framingham, MA 01702, USA; julia.maddalena@gmail.com; 7 Perennial Inc., Boulder, CO 80301, USA; 8 Department of Clinical and Experimental Medicine, University of Messina, 98125 Messina, Italy; 9 Division of Endocrinology and Metabolism, Faculty of Medicine, Siriraj Hospital, Mahidol University, Bangkok 10700, Thailand

**Keywords:** diabetes mellitus, risk prediction model, the Framingham Offspring Study, glucose, insulin, C-peptide

## Abstract

*Background*: Diabetes mellitus is a major cause of death and a significant risk factor for cardiovascular disease, kidney failure, neuropathy, and retinopathy. Our objectives were to develop a diabetes risk model and apply it to a large population. *Methods*: Non-diabetic adults in the Framingham Offspring Study (*n* = 2416) were followed for 10 years for new diabetes. At baseline, the fasting serum glucose, adiponectin, insulin, glycated albumin, total cholesterol, triglycerides (TG), and high-density lipoprotein cholesterol (HDL-C) were measured using standardized automated assays. Standard health information was collected. Diabetes risk prediction models were developed using logistic regression analysis and applied to a large population (*n* = 133,764). *Results*: In this prospective study, 166 subjects (6.9%) developed new-onset diabetes. Glucose, body mass index (BMI), log adiponectin, % log glycated albumin, parental diabetes, TG, and the use of cholesterol-lowering medications entered the model (C statistic: 0.924; 0.898, biochemical variables: 0.898, and fasting glucose: only 0.876). In the population in non-diabetic subjects (56.3) and prediabetic subjects (36.2%), the predicted 10-year diabetes risk rates were 0.4% and 5.5% with the biochemical model, respectively. Prediabetic and diabetic subjects were insulin-resistant compared to non-diabetic subjects, but only those with diabetes had significant reductions in their insulin production. *Conclusions*: The 10-year risk of diabetes can be accurately predicted and applied to large populations. Fasting glucose alone is diagnostic for diabetes and is an excellent predictor of future diabetes, with having prediabetes increasing the risk 6-fold. Insulin and C-peptide measurements are useful in diabetic subjects to detect decreased insulin production and the need for insulin therapy.

## 1. Introduction

The current criteria for the diagnosis of diabetes mellitus in the United States according to the American Diabetes Association (ADA) are a fasting serum glucose ≥ 126 mg/dL (7.0 mmol/L) and/or a glycosylated hemoglobin (HbA1c) value ≥ 6.5% or 48 mmol/mol, and/or 2 h glucose of ≥200 mg/dL or 11.1 mmol/L [1]. Subjects with diabetes are known to be at significantly higher risk of developing atherosclerotic cardiovascular disease (ASCVD), kidney disease, diabetic retinopathy, and neuropathy than subjects without diabetes [1,2]. Prediabetes has been defined as having a fasting serum glucose of 100–125 mg/dL and/or having an HbA1c value of 5.7–6.4% [1]. About one-third of the middle aged and elderly United States population has this condition [1].

According to the Centers for Disease Control (CDC), among the U.S. population overall, estimates for diabetes in 2021 were as follows: (1) 38.4 million people of all ages—or about 12% of the U.S. population—and 38.1 million aged 18 years or older (14.7% of all U.S. adults); (2) 8.7 million adults aged 18 years or older who met laboratory criteria for diabetes were not aware of having diabetes (3.4% of all U.S. adults), and 22.8% of all U.S. adults with diabetes [2]. The percentage of adults with diabetes increases with age, reaching 29.2% among those aged 65 years or older [2].

In their diabetes risk assessment point-count questionnaire, the ADA asks about the following: (1) age (1 point: 40–49; 2 points: 50–59; and 3 points: >60 years); (2) sex (1 point for male); (3) history of gestational diabetes (1 point for yes); (4) diabetes in a parent or sibling (1 point for yes); (5) history of hypertension (1 point for yes); (6) having a parent or sibling with type 2 diabetes (1 point for age); and (7) body mass index (BMI) (25–<30: 1 point; 30–<35: 2 points; and ≥35: 3 points) [1]. Those with a score of 5 points are at risk of developing diabetes and should be screened on an annual basis [1]. Based on this system, if you are over 60 years of age, male, and overweight, you have a high diabetes risk. This is also the case if you are a 65-year-old woman and are obese [1]. Additional risk factors identified by the ADA are as follows: (1) the use of glucocorticoids, statins, proprotein convertase subtilisin kexin 9 inhibitors, thiazide diuretics, some anti-psychotic medications, such as olanzapine, and medications for human immunodeficiency virus infection, (2) genetic causes for maturity-onset diabetes in the young (MODY) (<35 years of age) due to genetic mutations in the hepatic nuclear factor 1 and 4 alpha or HNF1A and HNF1B genes and the glucokinase or GCK gene, and (3) autoimmunity in childhood diabetes [1].

It is recommended that patients with prediabetes be placed on an intensive behavioral lifestyle intervention program modeled on the Diabetes Prevention Program to achieve and maintain a 7% loss of their initial body weight and to increase moderate–intensity physical activity (such as brisk walking) to at least 150 min/week [1]. The ADA has also provided excellent guidance on medications that are effective in the management of diabetes if lifestyle modification is insufficient to control it and obtain fasting glucose levels to <125 mg/dL and HbA1c to <7.0% [3]. Medications including metformin, pioglitazone, and glucagon-like peptide receptor agonists have all been shown to significantly decrease the development of diabetes in subjects with prediabetes versus placebos [3]. They also recommend screening every 3 years with C-peptide levels to assess for insulin deficiency [1].

Investigators have previously developed models for predicting the diabetes risk over 5–10 years using a variety of markers, including fasting glucose, BMI, high-density-lipoprotein cholesterol (HDL-C), parental diabetes history, and triglyceride levels [4,5,6,7,8,9,10,11,12,13,14]. Noble and colleagues reviewed data from 94 diabetes risk prediction models tested on a large number of individuals [11]. Some but not all of the risk models or scores had robust statistical properties (for example, good discrimination and calibration) and had been externally validated on a different population. Most authors described their score as “simple” or “easily implemented”, although few were specific about the intended users and under what circumstances.

Most recently, Liu and colleagues reviewed a total of 29,375 articles and examined 20 models from 24 studies in their meta-analysis [14]. The most common predictors of diabetes were age, body mass index, family history of diabetes, history of hypertension, and physical activity. They did not focus on biochemical variables. Discrimination and calibration were only reported in 79.2% and 4.2% of studies, respectively. The authors concluded that while numerous models are used to predict diabetes, screening in prediabetic populations would be most effective. They indicated there was no optimal model, and they did not recommend the use of the existing models for diabetes risk identification. They state that “future studies should focus on improving the clinical relevance and predictive performance of existing models” [11]. Our goals in this study were to (1) develop models for predicting diabetes in a prospective population study, and (2) apply these models to a large population.

## 2. Methods

### 2.1. Study Design and Participants

We studied participants in the Framingham Offspring Study (FOS), a long-term, community-based prospective observational study of risk factors for ASCVD. The FOS participants represent the offspring and spouses of the original Framingham Heart Study cohort, as previously described [5]. During the sixth biennial examination, 3188 participants (52.7% female, almost entirely Caucasian) were available for study, underwent a standardized medical history and physical examination, and had blood drawn for serum and plasma analysis [6]. Information on medication use and parental history of diabetes was obtained at baseline [6,7,15,16,17]. The physical examination included the measurement of blood pressure, height, weight, and waist circumference. Those with a blood pressure ≥ 130/80 mmHg and/or receiving treatment for hypertension were considered to have hypertension.

We excluded 19.0% of subjects from this study since they were (1) not seen at the 10-year follow-up examination (by far the largest group), and/or (2) did not have frozen plasma available for study, and/or (3) were not fasting for at least 8 h. In addition, 6.4% of subjects were classified as diabetic at baseline; they were also excluded. The remaining 2416 subjects (55.3% female) were available for study and were followed from baseline for a median of 9.9 years. Being classified as developing new diabetes during follow-up required either the new use of oral hypoglycemic agents or insulin or a fasting glucose level ≥ 126 mg/dL, or both, at any follow-up visit. This situation occurred in 166 subjects (6.9%, classified as converters) (Table 1). Analyses were carried out comparing these subjects (converters) with those that did not become diabetic during follow-up (non-converters; *n* = 2250, 93.1%) (Table 1 and Table 2). Informed consent was obtained from all subjects using protocols and consent forms approved by the institutional review boards from Boston University Medical Campus (H-39410, Date of Approval: 11 January 2019).

After defining the predictive equations for diabetes risk, subsequent clinical population studies were carried out as a retrospective analysis on a total of 135,929 subjects throughout the United States whose serum samples were sent by their physicians (gel separator blood collection tubes shipped on ice packs by overnight courier service) to a national clinical reference laboratory (Boston Heart Diagnostics, Framingham, MA, USA) for cardiovascular risk assessment. Only data from fasting subjects were used. All data were anonymized prior to analysis. Such analyses are exempt from institutional review board review (exemption 4, https://grants.nih.gov/policy/humansubjects.htm (accessed on 18 November 2021), open education resource website 45 CFR 46·104(d)). Of these 135,929 subjects, 1.6% were excluded because they had been diagnosed with diabetes and were on insulin therapy. Of the remaining 133,774 subjects, based on testing over a 3-year period of time, 56.3% were classified as non-diabetic (fasting glucose: <100 mg/dL), 36.2% were classified as prediabetic (fasting glucose: 100–125 mg/dL), and 7.5% were classified as diabetic (fasting glucose: ≥126 mg/dL or receiving diabetes medication, but not insulin) (Table 3). It is important to emphasize that these subjects may not be representative of the entire United States population since their samples were sent to our specialty laboratory by their healthcare providers. Our laboratory specializes in cardiovascular disease prediction and prevention.

### 2.2. Laboratory Measurements

For FOS participants, fasting plasma samples stored at −80 °C and never thawed were used for standardized laboratory analyses at Tufts University using a Hitachi 911 analyzer, as previously described, with with- and between-run coefficients of variation of <3.0% [6,15,16,17]. Measurements consisted of total cholesterol, HDL-C, and triglycerides (Roche Diagnostics, Indianapolis, IN, USA), adiponectin (immunoassay, Otsuka Pharmaceutical Corporation, Tokyo, Japan), glycated albumin (Asahi-Kasei, Tokyo, Japan), and insulin (Kamiya Biomedical, Seattle, WA, USA). Fasting glucose and high-sensitivity C-reactive protein (hs-CRP) were measured using assays as previously described [6,18].

The laboratory tests performed on fasting serum samples sent by physicians to Boston Heart Diagnostics included the following: (1) glucose, (2) insulin, (3) C-peptide, (4) hs-CRP, (5) direct LDL-C, (6) HDL-C (assay kits from Roche Diagnostics, Indianapolis, IN, USA), (7) adiponectin, and (8) glycated serum protein (GSP) (assay kits from Diazyme Laboratories, Poway, CA, USA). HbA1c analysis was run on red blood cell samples (assay kits from Roche Diagnostics, Indianapolis, IN, USA). All assays had within- and between-run coefficients of variation of <4.0%.

For both populations, the homeostasis model of insulin resistance (HOMA-IR) was calculated as [fasting insulin (µU/mL) × fasting plasma glucose (mg/dL)]/405, and the homeostasis model of insulin production (HOMA-β) was calculated as [360 × fasting insulin (µU/mL)]/[fasting plasma glucose (mg/dL) − 63], as previously described [19].

### 2.3. Statistical Analysis

For the FOS data, we calculated the means and standard deviations of each continuous variable and the percentage of each categorical variable for converters and non-converters. We compared the mean or proportion difference of the variables between converters and non-converters using *t*-tests for continuous variables and chi-square tests for categorical variables. For non-normally distributed variables (fasting adiponectin, glycated serum albumin, insulin, hs-CRP, and triglycerides), data are presented as median values with the interquartile range. We compared differences in these variables between converters and non-converters using Kruskal–Wallis tests. The values of these variables were log-transformed prior to multivariate analysis. *p* values < 0.05 were considered statistically significant. For converters and non-converters, the percent difference for all variables comparing the mean or median values are provided. The definition of metabolic syndrome used for both populations was having three of the following five characteristics: (1) fasting glucose ≥100 mg/dL, (2) BMI > 25 kg/m^2^, (3) fasting triglycerides > 150 mg/dL, (4) blood pressure ≥130/80 mmHg, and (5) HDL-C < 40 mg/dL in men and <50 mg/dL in women.

Logistic regression analysis was performed to predict incident diabetes. Odds ratios and 95% confidence intervals to estimate the relative risk are presented (Table 2). Logistic regression models are presented using (1) fasting glucose only (Model 1), (2) the four significant biochemical variables of fasting glucose, log glycated albumin, log adiponectin, and log triglycerides (Model 2), and (3) the full model which included the biochemical variables as well as BMI, parental history of diabetes, and use of cholesterol-lowering medications (93% statins) (Model 3). Models were assessed using the C statistic and the area under the receiver operating characteristic curve. Between-model comparisons were evaluated by ranking participant risk by decile and performing an analysis on the estimates, as described by Hosmer and Lemeshow [20].

For the Boston Heart Diagnostics population, clinical and biochemical variables in non-diabetic, prediabetic, and diabetic subjects are expressed as median values with the interquartile range (25–75th percentile) value provided. Variables that were not normally distributed, including adiponectin, glycated albumin, hs-CRP, insulin, and triglycerides, were log-transformed before statistical analysis. Categorical variables are provided by percentage. All variables between subjects that were non-diabetic, prediabetic, or diabetic were compared using non-parametric Kruskal–Wallis testing. To predict the diabetes risk in non-diabetic subjects and prediabetic subjects in the Boston Heart Diagnostics population, the biochemical model, Model 2, was used. The percent difference for all variables comparing the median values of the diabetic and control subjects are provided. The HOMA-IR value was calculated using the formula [fasting insulin (µU/mL) × fasting plasma glucose (mg/dL)]/405. The HOMA-β was calculated as follows: [360 × fasting insulin (µU/mL)]/[fasting plasma glucose (mg/dL) − 63] [19].

## 3. Results

### 3.1. Diabetes Prediction

Data on the Framingham Offspring Study participants who did not develop diabetes (*n* = 2250), as compared to those who developed diabetes (*n* = 166; 6.9% over the follow-up period), are shown in Table 1. At baseline, 466 subjects (19.3%) had prediabetes (fasting serum glucose of 100–125 mg/dL and not on treatment for diabetes). Of the 466 subjects with prediabetes, 97 (20.9%) developed diabetes (fasting glucose ≥ 126 mg/dL) over the follow-up period. A total of 80.2% of these prediabetic subjects had metabolic syndrome. Therefore, the risk of developing diabetes in the prediabetic subjects in the FOS was about 6-fold higher than in subjects with a baseline fasting glucose of <100 mg/dL.

All variables shown in Table 1 were significantly different in converters compared to non-converters. In terms of percent differences between converters and non-converters, the order of variables from the greatest percentage difference to the least percentage difference was as follows: (1) cholesterol treatment, (2) hs-CRP, (3) parental diabetes, (4) being hypertensive, (5) fasting insulin, (6) fasting triglycerides, (7) gout treatment, (8) low HDL-C, (9) adiponectin, (10) sex, (11) fasting glucose, (12) BMI, (13) waist circumference, and (14) age.

In the logistic regression analysis, fasting glucose was by far the most important variable in predicting diabetes risk of the 14 variables tested. As shown in Table 2, we obtained a beta estimate of 0.1644 (0.010) with an odds ratio of 1.179 (*p* < 0.001) for every 1 mg/dL increase in fasting glucose and a C statistic of 0.876. These data indicate that fasting glucose alone is highly predictive of the development of future diabetes. The subjects who did not develop diabetes had a mean fasting glucose at baseline of 95.6 mg/dL, while those who became diabetic over the follow-up period had a mean fasting glucose of 110.9 mg/dL (odds ratio: 18.0, *p* < 0.001). In the biochemical model, Model 2 (Table 2), the following biochemical variables entered the model: (1) fasting glucose, (2) log glycated serum albumin, (3) log adiponectin, and (4) fasting triglycerides. The addition of these parameters to fasting glucose significantly improved the C statistic to 0.898 (about a 3% gain in the absolute C statistic value) and the accuracy of the diabetes risk prediction (*p* < 0.001). In the full model (Model 3), with the use of all four biochemical variables and the addition of BMI, parental history of diabetes, and cholesterol-lowering treatment (Table 2), the C statistic was further increased to 0.924, representing another approximate absolute 3% gain in the C statistic over the biochemical model and a 5.5% absolute gain over just using fasting glucose (*p* < 0.001). None of the other parameters (total cholesterol, HDL-C, insulin, or hs-CRP) were significant in the biochemical model or the complete model. 

The equations generated for use to calculate the 10-year risk of developing diabetes are as follows:

For fasting glucose only: X = −24.1468 + (fasting glucose × 0.164);

For the biochemical model only: X = −24.1468 + (fasting glucose × 0.152) + (log % glycated albumin × 2.882) + (log adiponectin × −0.1.075) + (fasting triglyceride × 0.00418);

For the full model: X = −30.1032 + (fasting glucose × 0.1578) + (BMI × 0.0943) + (log % glycated albumin × 3.7174) + (log adiponectin × −0.9768) + parental history of diabetes × 0.6662)) + (fasting triglycerides × 0.00317).

For the above equations, fasting glucose is in mg/dL, BMI is in kg/m^2^, glycated albumin is in %, adiponectin is in µg/mL, and parental history of diabetes is 1 = yes, 0 = no. To convert glycated serum protein to percent glycated albumin, the following formula is used: % glycated albumin = [(0.182 × glycated serum protein in µmol/L)/total albumin in g/dL) + 2.9]. X, the probability (P) of developing diabetes over 10 years, is plugged into the equation P = 1/(1 + ex), which is the % risk, and X is the value obtained from either of the above two equations. The C statistics for each model are provided in Table 2.

### 3.2. Large Population Analysis

Data on the large Boston Heart Diagnostics population are presented in Table 3 and Table 4. The percentage of subjects receiving cholesterol-lowering therapy was 12.1% in the non-diabetic population, 17.5% in the prediabetic population, and 31.2% in the diabetic population. In terms of comorbidities, the percentage of subjects with cardiovascular disease (heart disease or stroke) was 4.1% in the non-diabetic population, 11.1% in the prediabetic population, and 24.5% in the diabetic population. In the prediabetic subjects, 90.3% had criteria for metabolic syndrome. The greatest difference between the diabetic and control subjects was the HOMA-IR, which was 305.6% higher in subjects with diabetes, indicating marked insulin resistance. Prediabetic subjects had a median HOMA-IR value that was 94.4% higher than that of the controls. In contrast, the HOMA-β was 42.1% lower in the diabetic subjects than in the controls, but only 2.7% lower in the prediabetic subjects than in the controls. While almost all the diabetic subjects were insulin-resistant, about 25% also had evidence of decreased insulin production and relative insulin deficiency. After the HOMA-IR, the greatest percent differences observed for the other 13 variables examined (not including the HOMA-IR and HOMA-β), in order, comparing diabetic subjects with non-diabetic subjects, were as follows: (1) hs-CRP level (+127.3%); (2) insulin level (+112.5%); (3) C-peptide level (+81.0%); (4) glucose level (+71.1%); (5) male sex (+64.0%); (6) triglyceride level (+61.3%); (7) GSP level (+43.9%); (8) adiponectin level (–34.1%); (9) HbA1c level (+30.9%); (10) HDL-C level (–25.9%); (11) BMI (+18.5%); (12) age (+15.4%); and (13) LDL-C level (–8.5%).

## 4. Discussion

Multiple studies have developed models for predicting diabetes risk, with C statistic values ranging from 0.75 to 0.91 [7,8,9,10,11,12,13]. The 5-year risk prediction models developed using the Inter99 cohort and the combined Inter99 and Botnia cohorts included fasting glucose, fasting insulin, adiponectin, hs-CRP, ferritin, HbA1c, interleukin-2 receptor antigen, age, and sex, resulting in C statistics of 0.85 and 0.79, respectively [9,10]. The prior Framingham model for diabetes prediction over 5–7 years, which included glucose, BMI, parental diabetes, triglycerides, HDL-C, and blood pressure, had a final C statistic of 0.85 [5]. In our analysis of the Framingham Offspring cohort, fasting glucose alone was by far the most important predictor (C statistic: 0.876). When we added adiponectin, glycated albumin, triglycerides, parental diabetes history, BMI, and use of cholesterol-lowering medication, which is known to increase diabetes risk, the prediction was significantly improved. The C statistics were 0.898 for the biochemical variables only and 0.924 for the full model.

Since the development of insulin immunoassays by Yalow and Berson, it has become clear that type 2 diabetes is often associated with both insulin resistance and relative deficiency [19,21,22,23,24,25]. These findings have been facilitated by the development of equations for the calculation of the HOMA-IR and HOMA-β [19,22,23,24,25]. Investigators from the San Antonio Heart Study and the Framingham Offspring Study have documented that an oral glucose tolerance test did not significantly improve diabetes prediction [4,5]. In their analyses, as well as in our own, adding the fasting insulin levels or the HOMA-IR also did not improve the C statistic significantly. However, the measurement of the fasting insulin and the calculation of the HOMA-IR and HOMA-β were essential for identifying subjects with diabetes requiring insulin therapy because of deficiency with low production, as seen in our large population. In our view, it is in diabetic subjects that the measurement of insulin or C-peptide has the greatest value, and this approach has now been emphasized by the American Diabetes Association [1,26]. This measurement, in our view, is essential, along with the fasting glucose, to determine whether there is a relative insulin deficiency and whether such patients require insulin therapy.

Our study indicates that diabetes risk prediction is most valuable in prediabetic subjects, and that fasting glucose alone is an excellent predictor of diabetes in this group. In our Framingham cohort, prediabetic subjects had a 10-year diabetes risk of about 21%, 3-fold higher than that in the general non-diabetic population and 6-fold higher than that in those with fasting glucose values < 100 mg/dL. It should be noted that adiponectin, glycated albumin, and fasting triglycerides add to risk prediction, fasting glucose alone provides very strong prediction, and this parameter is available in any clinical setting.

Type 2 diabetes has long been thought to have heterogenous causes, even though epidemiological studies uniformly show a tight relationship with overnutrition. Taylor has postulated that “the twin cycle hypothesis postulated that interaction of self-reinforcing cycles of fat accumulation inside the liver and pancreas, driven by modest but chronic positive calorie balance, could explain the development of type 2 diabetes” [27]. This hypothesis predicted that substantial weight loss would bring about a return to the non-diabetic state, permitting observation of the pathophysiology determining the transition. These changes were postulated to reflect the basic mechanisms of causation in reverse. A series of studies over the past 15 years has elucidated these underlying mechanisms. This knowledge has led to the successful implementation of national programs for the remission of type 2 diabetes. In Taylor’s view, type 2 diabetes has a homogenous cause expressed in genetically heterogenous individuals. However, we have noted that the prevalence of diabetes is similar in the Kyushu–Okinawa Population Study to that in the FOS, but the BMI levels are much lower, as are the insulin levels and HOMA-IR, and there is a higher prevalence of low HOMA-β in Japan as compared to the FOS [28,29]. Therefore, in some patients, weight loss alone may not reverse diabetes, even though powerful new weight loss medications have now become available.

### Study Strengths and Weaknesses

A strength of these studies is the large number of subjects in our population studies and the many parameters that were assessed. The limitations of our studies are the relatively small sample size of our Framingham cohort and the lack of clinical information and lack of long-term follow-up for the diabetic subjects identified with decreased insulin production in our large population study. Another limitation is that the study participants were almost all Caucasian. Therefore, these data may not be valid for African Americans, Hispanic subjects, or subjects of Asian origin. In addition, the large population may not be representative of the entire United States population.

## 5. Conclusions

Our studies are consistent with the following conclusions: (1) diabetes risk prediction is useful in subjects with prediabetes; (2) fasting glucose alone is an excellent predictor of diabetes risk; (3) prediabetic subjects should have an annual measurement of their fasting glucose; and (4) the measurement of the fasting glucose and insulin and the calculation of the HOMA-IR and HOMA-β are important for identifying diabetic subjects with low insulin production who may require insulin therapy to control their glucose and HbA1c levels.

## Figures and Tables

**Table 1:** Characteristics of non-converter and converter subjects at baseline *.

Parameter ^†^	Non-Converters (*n* = 2250)	Converters (*n* = 166)	% Difference
Demographics			
Age, years	57.9 (9.6)	59.6 (8.6) ^c^	+2.9%
Sex, % female	55.7%	43.4% ^b^	–22.1%
Body mass index, kg/m^2^	27.3 (4.8)	31.6 (5.5) ^a^	+15.8%
Waist, cm	95.9 (12.9)	107.4 (12.1) ^a^	+12.0%
Clinical Treatment			
Parental diabetes, %	22.3%	37.6% ^a^	+68.6%
Hypertensive, %	35.9%	55.4% ^a^	+54.3%
Cholesterol treatment, %	10.0%	22.0% ^b^	+120.2%
Uric acid treatment, %	1.29%	1.81% ^c^	+40.3%
Metabolism			
Fasting glucose, mg/dL	95.8 (9.1)	110·9 (8.9) ^a^	+15.8%
Glycated albumin, % ^‡^	14.1 (1.8)	14.5 (1.8) ^a^	+2.8%
Log glycated albumin	2.64 (0.10)	2.68 ^a^ (0.11) ^a^	
Insulin, microU/mL ^‡^	10.1 (5.4)	15.4 (9.1) ^a^	+52.5%
Log insulin	2.36 (0.40)	2.72 (0.42) ^a^	
Adiponectin, ug/mL ^‡^	12.0 (8.8)	8.2 (4.5) ^a^	–31.7%
Log adiponectin	2.49 (0.51)	2.11 (0.42) ^a^	
Inflammation			
hs-CRP, mg/L ^‡^	1.88 (1.71)	3.38 (2.82) ^b^	+79.8%
Log C-reactive protein	0.67 (1.14)	1.23 (1.11) ^a^	
Lipids			
Triglycerides, mg/dL ^‡^	110 (77)	155 (97) ^a^	+40.9%
Log triglycerides	4.70 (0.49)	5.02 (0.48) ^a^	
HDL cholesterol, mg/dL	52.7 (15.8)	43.4 (11.5) ^a^	–17.6%

* Median follow-up was 9.9 years. All subjects were assessed after an overnight fast. For non-converters, 14.3% were smokers versus 16.4% of converters (ns). New development of coronary heart disease was 5.7% in non-converters and 12.1% in converters, a 2.1-fold difference (*p* < 0.0001). The calculated value for the homeostasis model assessment of insulin resistance (HOMA-IR) was 63.0% higher in converters than non-converters (*p* < 0.0001), and for insulin production, it was 31.2% lower (*p* < 0.0001). ^†^ All parameters are expressed as means (±SDs) except those marked as ^‡^, which are expressed as medians (interquartile ranges). ^a^ *p* < 0.0001; ^b^ *p* < 0.001; ^c^ *p* < 0.05.

**Table 2:** Logistic regression models for diabetes prediction.

Parameter	Beta Value	Error	Odds Ratio	*p* Value	*C* Statistic (Sum)
Fasting Glucose—Model 1					
Fasting glucose, mg/dL	0.1644	0.010	1.179	<0.001	0.876 ***
Biochemical Model—Model 2					
Fasting glucose, mg/dL	0.1520	0.011	1.164	<0.0001	0.898 ***
Log adiponectin	–1.0750	0.215	0.3413	<0.0001	***
Fasting triglycerides, mg/dL	0.0042	0.001	1.004	0.0003	**
Log glycated albumin, %	2.8820	0.859	17.85	0.0008	**
Full Model—Model 3					
Fasting glucose, mg/dL	0.1578	0.014	1.171	< 0.0001	0.924 ***
Body mass index, kg/m^2^	0.0943	0.022	1.099	< 0.0001	***
Log adiponectin, %	–0.9768	0.258	0.377	0.0002	**
Log glycated albumin	3.7174	1.063	41.16	0.0005	**
Parental diabetes, y/*n*	0.6662	0.238	1.947	0.0051	*
Fasting triglycerides, mg/dL	0.0032	0.001	1.003	0.0227	*
Cholesterol treatment, y/*n*	0.5706	0.285	1.769	0.0454	*

The parameters age, sex, systolic blood pressure, blood pressure treatment, waist circumference, HDL-C, insulin, calculated insulin resistance, uric acid, and uric acid treatment were not significant after the above parameters were placed in the model. The odds ratio represents the value per one unit change in the parameter shown. *** *p* < 0.0001, ** *p* < 0.001, * *p* < 0.05 (converters versus non-converters). The following equations were developed for calculating the 10-year risk of developing diabetes: (1) full model: X = [−30.1032/fasting glucose (mg/dL) × 0.1576]/[BMI (mg/kg/m^2^) × 0.0943)]/[log % glycated albumin × 3.7174]/log adiponectin (µg/mL) × −0.9769}.

**Table 3:** Characteristics of prediabetic and diabetic subjects compared with non-diabetic subjects for variables used in model.

Parameter	No Diabetes *n* = 75,271 (56.3%)		Prediabetes *n* = 48,455 (36.2%)		Diabetes *n* = 10,038 (7.5%)		% Difference, vs. Non-Diabetic Subjects
	N	Median (IQR)	N	Median (IQR)	N	Median (IQR)	
Demographics							
Age, years	75,271	52.0 (22.0)	48,455	59.0 (17.0)	10,038	60.0 (17.0) *	+15.4%
Females	48,476	52.0 (22.0)	22,977	59.0 (17.0)	4175	60.0 (18.0) *	+15.4%
Males	26,795	52.0 (23.0)	25,478	58.0 (18.0)	5863	60.0 (17.0) *	+15.4%
Sex							
Females	48,476	64.4%	22,977	47.4%	4175	41.6% *	–35.4%
Males	26,795	35.6%	25,478	52.6%	5863	58.4% *	+64.0%
BMI, kg/m^2^	12,794	27.0 (7.0)	11,758	30.0 (8.0)	801	32.0 (9.0) *	+18.5%
Females	8575	26.0 (8.0)	5605	30.0 (10.0)	331	34.0 (10.0) *	+30.8%
Males	4219	28.0 (7.0)	6153	30.0 (6.0)	470	31.0 (7.0) *	+10.7%
Glucose, mg/dL	75,271	90.0 (10.0)	48,455	106.0 (10.0)	10,038	154.0 (57.0) *	+71.1%
Females	48,476	90.0 (9.0)	22,977	105.0 (9.0)	4175	153.0 (56.0) *	+70.0%
Males	26,795	92.0 (9.0)	25,478	106.0 (10.0)	5863	155.0 (57.0) *	+68.5%
Adiponectin, μg/mL	75,271	12.6 (9.3)	48,455	10.1 (7.6)	10,038	8.3 (6.3) *	–34.1%
Females	48,476	14.6 (9.6)	22,977	12.1 (8.6)	4175	9.7 (7.4) *	–33.6%
Males	26,795	9.4 (6.4)	25,478	8.6 (5.9)	5863	7.5 (5.4) *	–20.2%
Glycated serum protein, μmol/L	75,269	199 (53)	48,454	205 (59)	10,038	299 (62) *	+50.3%
Females	48,476	198 (55)	22,976	200 (60)	4175	285 (98) *	+43.9%
Males	26,793	202 (52)	25,478	210 (58)	5863	308 (63) *	+52.5%
Diabetes risk (calculated, biochemical)	75,271	0.4 (0.6)	48,455	5.5 (12.1)	10,038	100.0 *	+150%
Females	48,476	0.3 (0.5)	22,977	4.2 (8.9)	4175	100.0 *	+233%
Males	26,795	0.6 (1.0)	25,478	7.0 (14.7)	5863	100.0 *	+67%
Triglycerides, mg/dL	75,271	93 (67.0)	48,455	116 (80.0)	10,038	150 (114) *	+61.3%
Females	48,476	89 (60.0)	22,977	115 (77.0)	4175	151 (107) *	+69.7%
Males	26,795	103 (75.0)	25,478	116 (83.0)	5863	149 (118) *	+44.7%

* *p* < 0.001 based on non-parametric Kruskal-Wallis test comparing non-diabetic subjects with prediabetic subjects with diabetic subjects.

**Table 4:** Characteristics of prediabetic and diabetic subjects compared with non-diabetic subjects for non-model variables.

Parameter	No Diabetes *n* = 75,271 (56.3%)		Prediabetes *n* = 48,455 (36.2%)		Diabetes *n* = 10,038 (7.5%)		% Difference, vs. Non-Diabetic Subjects
	N	Median (IQR)	N	Median (IQR)	N	Median (IQR)	
HbA1c, %	72,980	5.5 (0.5)	45,176	5.7 (0.5)	9599	7.2 (1.9) *	+30.9%
Females	47,206	5.4 (0.5)	21,540	5.7 (0·5)	3989	7.2 (1.8) *	+33.3%
Males	25,774	5·5 (0·4)	23,636	5.7 (0·6)	5610	7.2 (1.9) *	+30.9%
Insulin, microU/mL	73,624	8.0 (8.0)	45,176	13.0 (12.0)	9940	17.0 (18.0) *	+112.5%
Females	47,420	8.0 (7.0)	21,477	13.0 (12.0)	4130	18.0 (18.0) *	+125.0%
Males	26,204	9.0 (8.0)	23,699	13.0 (12.0)	5810	17.0 (19.0) *	+88.9%
HOMA-IR	73,460	1.8 (1.8)	45,069	3.5 (3.3)	9781	7.3 (8.1) *	+305.6%
Females	47,331	1.7 (1.6)	21,421	3.6 (3.3)	4042	7.6 (8.0) *	+347.1%
Males	26,129	2.0 (2.0)	23,648	3.5 (3.3)	5739	7.2 (8.2) *	+260.0%
HOMA-β	73,572	111 (102)	45,176	108 (95)	9940	64 (81) *	–42.3%
Females	47,390	109 (98)	21,477	110 (96)	4130	68 (83) *	–37.6%
Males	26,182	115 (111)	23,699	107 (96)	5810	62 (80) *	–46.1%
C-peptide, ng/mL	10,322	2.1 (1.3)	4897	3.2 (1.8)	1394	3.8 (2.4) *	+81.0%
Females	6597	2.0 (1.1)	2310	3.2 (1.8)	531	3.8 (2.5) *	+90.0%
Males	3725	2.3 (1.5)	2587	3.2 (1.8)	863	3.8 (2.3) *	+65.2%
hs-CRP, mg/L	72,717	1.1 (2.4)	45,569	1.6 (3.1)	9765	2.5 (4.4) *	+127.3%
Females	46,937	1.2 (2.7)	21,723	2.2 (4.1)	4052	3.6 (6.0) *	+200.0%
Males	25,780	1.0 (1.9)	23,846	1.2 (2.3)	5713	1.9 (3.3) *	+90.0%
LDL-C, mg/dL	73,824	117 (51.0)	46,550	117 (54.0)	9966	107 (57.0) *	–8.5%
Females	47,539	117 (49.0)	22,127	121 (53.0)	4144	114 (59.0) *	–2.6%
Males	26,285	117 (54.0)	24,423	113 (55.0)	5822	102 (55.0) *	–12.8%
HDL-C, mg/dL	74,272	58 (24.0)	46,978	51 (22.0)	10,017	43 (17.0) *	–25.9%
Females	47,793	64 (25.0)	22,306	57 (22.0)	4161	49 (19.0) *	–23.4%
Males	26,479	49 (19.0)	24,672	46 (18.0)	5856	40 (15.0) *	–18.4%

* *p* < 0.001 based on non-parametric Kruskal–Wallis test comparing non-diabetic subjects with prediabetic subjects with diabetic subjects.

## Data Availability

The data from the Framingham Heart Study are available from the Framingham Offspring Study upon request; the initial contact would be Dr. Li-Ching Liu (ctliu@bu.edu). The Boston Heart Diagnostics data (anonymized) are available from Dr. Margaret R. Diffenderfer upon request (Margaret.diffenderfer@bostonheart.eurofinsus.com).

## Abbreviations

ASCVD	Atherosclerotic cardiovascular disease
BMI	Body mass index
FOS	Framingham Offspring Study
GSP	Glycated serum protein
HbA1c	Glycosylated hemoglobin
HDL-C	High-density lipoprotein cholesterol
HOMA-IR	Homeostasis model of insulin resistance
HOMA-β	Homeostasis model of insulin production
hs-CRP	High-sensitivity C-reactive protein
LDL-C	Low-density lipoprotein cholesterol
